# Geriatric Patient-Aligned Care Teams in Department of Veterans Affairs: How Are They Structured?

**DOI:** 10.3390/geriatrics3030046

**Published:** 2018-08-01

**Authors:** Jennifer L. Sullivan, Marlena H. Shin, Omonyêlé L. Adjognon, Kenneth Shay, Kimberly Harvey, Orna Intrator, Enzo Yaksic, Jennifer Moye, Samantha Solimeo

**Affiliations:** 1 Center for Healthcare Organization and Implementation Research, VA Boston Healthcare System, 150 S. Huntington Ave (152M), Boston, MA 02130, USA; marlena.shin@va.gov (M.H.S.); omonyele.adjognon@va.gov (O.L.A.); kimberly.harvey@va.gov (K.H.); jennifer.moye@va.gov (J.M.); 2 Department of Health Law, Policy and Management, Boston University School of Public Health, 715 Albany St, Talbot Building, Boston, MA 02118, USA; 3 US Department of Veterans Affairs, Office of Geriatrics and Extended Care (GEC) (10NC4), Washington, DC 20420, USA; kenneth.shay@va.gov; 4 Department of Public Health Sciences, University of Rochester, 265 Crittenden Blvd, Rochester, NY 14642, USA; orna.intrator@va.gov; 5 VA Geriatrics and Extended Care Data Analysis Center (GEC DAC), Canandaigua VA Medical Center, 400 Fort Hill Ave, Canandaigua, NY 14424, USA; 6 Massachusetts Veterans Epidemiology Research and Information Center (MAVERIC), VA Boston Healthcare System, 150 S. Huntington Ave (152M), Boston, MA 02130, USA; enzo.yaksic@va.gov; 7 New England Geriatric Research Education and Clinical Center (GRECC), VA Boston Healthcare System, 150 S. Huntington Ave (152M), Boston, MA 02130, USA; 8 Center for Comprehensive Access & Delivery Research & Evaluation Center and the Veterans Integrated Service Network (VISN) 23 Patient Aligned Care Team (PACT) Demonstration Laboratory, Iowa City VA Health Care System, Iowa City, IA 52246, USA; samantha.solimeo@va.gov; 9 Department of General Internal Medicine, University of Iowa Carver College of Medicine, Iowa City, IA 52242, USA

**Keywords:** patient-centered medical homes, chronic disease, implementation, geriatrics

## Abstract

Geriatric Patient-Aligned Care Teams (GeriPACT) were implemented in the Department of Veterans Affairs (VA) (i.e., Patient-Centered Medical Homes for older adults) to provide high quality coordinated care to older adults with more risk of negative health and psychosocial outcomes. The objectives of this paper are: (1) to present data on GeriPACT structural characteristics; and (2) to examine a composite measure of GeriPACT model consistency. We utilized a web survey targeting 71 physician leads resulting in a 62% response rate. We found GeriPACTs employed a range of staffing, empanelment, clinic space, and patient assignment practices. The mean value of the GeriPACT consistency measure was 2.03 (range: 1–4) and 6.3% of facilities were considered consistent to the GeriPACT model. We observed large variation in GeriPACT structure and in model consistency. More research is needed to understand how these variations are related to processes and outcomes of care.

## 1. Introduction

The Patient-Centered Medical Home (PCMH) model was developed to provide more timely access, coordination and patient-centered care in primary care settings. To date, the evidence regarding the success of PCMH has been mixed, although some research has shown PCMH implementation was related to slightly better clinical outcomes [1,2,3]. However successful its implementation, PCMH was not designed with the unique needs of an older adult population in mind. The multiple interacting cognitive, functional, psychosocial, and medical challenges of older adults may surmount the capacity of a typical PCMH [4]. For example, about three-quarters of Americans who are 65 and older have multiple chronic conditions that require ongoing medical attention or limit activities of daily living [5] In addition, older patients can experience geriatric syndromes that require more intensive care management, such as dementia and frailty. Older adults also deal with psychosocial issues such as living alone, and/or geographic isolation, in relationship to family or in terms of rurality, which tend to negatively impact older adults in ways that they might not have imagined at earlier ages. 

As the medical complexity and psychosocial needs in older adults increase, the risk for negative outcomes, such as hospitalization or need for supportive care, also increases. Given the unique needs and considerable growth of this specific population (43.1 million in U.S. in 2012 to 83.7 million in 2050 [6]), tailoring the PCMH model for older adults may help to mitigate negative outcomes by providing: (1) better care coordination within an interdisciplinary care team; (2) better access to community resources; (3) longer appointments to address the needs of complex patients; and (4) access to specific resources, such as having a pharmacist on the team to manage polypharmacy concerns and increased social work support to address psycho-social issues. A PCMH model designed to meet the needs of older adults also may increase patient and provider satisfaction, and reduce costs and healthcare utilization. 

In recognition of the unique primary care needs of the older adult population (age 65 or greater), the Department of Veterans Affairs (VA) implemented a specialized PCMH called the Geriatric Patient Aligned Care Team (GeriPACT) in 2010. Approximately 51% of the current active VA patient population is comprised of older adults; in fact, it is projected that just the patient population of Veterans aged 75 and older alone will increase by approximately 38%. GeriPACTs deliver focused care to Veterans with multiple chronic diseases as well as coexisting cognitive, functional, and psychosocial and provides these patients and their caregivers with a single point-of-contact for intensive, interdisciplinary Geriatric Primary Care [7]. GeriPACT differs from other VA primary care teams (e.g., PACTs) in several ways: (1) the GeriPACT includes a social worker and pharmacist on the primary care team; (2) GeriPACT providers have a reduced panel size (2/3 the size of a PACT panel per full time provider); and (3) team members must have Geriatrics training or advanced, mentored experience in providing care to older adults [8]. VA has released a handbook outlining guidelines and criteria for the GeriPACT Model [7]. 

In fiscal year (FY) 2015, approximately 1.5 million enrolled Veterans aged 65 and older were found to have a high Jen Frailty Index (JFI) score (i.e., claims-based risk-adjustment measure that serves as a marker of functional impairment based on 13 condition categories found to be significantly related to need for long-term care services) [9]. Of these, an estimated 60,000 patients (<0.01%) were receiving care through GeriPACT which, since its initiation in 2010, had grown to a service being offered in approximately 66 (46.8%) of VA’s approximately 141 facilities. This suggests that Veterans could be much more widely served using the GeriPACT model of care. In addition, there is wide variation in the rate of GeriPACT adoption across facilities To date, little research has been conducted to understand how VA medical centers (VAMCs) are structuring their GeriPACT programs to serve older Veterans and the extent to which GeriPACTs are consistent with the structural characteristics outlined in the VA GeriPACT Handbook [7]. Structural characteristics are important to assess because they control how providers and patients in a healthcare system interact and are related to quality of care within a facility or system [10]. Thus, the objective of this paper is to present our findings on the structural characteristics of GeriPACTs across a sample of 44 VAMCs by: (1) depicting data on individual GeriPACT structural characteristics; and (2) examining a composite measure to assess consistency with the GeriPACT model [7]. VA is an ideal setting in which to examine the PCMH model because it is a pioneer in adopting this model, having initiated the program system-wide eight years ago. Our findings can be used to strengthen the GeriPACT model as it continues to expand and can shape VA policy. In addition, given there has not been much uptake of the PCMH model for older adults in the private sector, our findings can help guide non-VA providers on ways to structure or tailor the model. 

## 2. Materials and Methods

We conducted an observational study of GeriPACT structural characteristics and consistency to the GeriPACT model as part of a non-research evaluation in collaboration with the VA Office of Geriatrics and Extended Care. The VA Boston Healthcare System’s Research and Development Committee determined that the work conducted for this study was quality improvement and not research.

### 2.1. Survey Development

To assess the GeriPACT structural characteristics across VAMCs, we developed a survey based on the critical program components described in the GeriPACT Handbook [7]. Our survey incorporated questions from a previously administered survey regarding geriatrics service delivery in VA that was sponsored by the Office of Geriatrics and Extended Care. We have included the survey questions used as part of this paper in the Supplemental Material. The survey was then programmed using Verint^®^ Enterprise Feedback Management, a VA-approved software package.

### 2.2. Measures

Structural characteristics. The survey requests data pertaining to team structural characteristics such as: GeriPACT staffing (i.e., full-time employment equivalent); allocation of clinical space; panel size; patient enrollment procedures; conditions managed; services provided (e.g., use of comprehensive geriatric assessments, having a patient educator, having a case manager, and having a collaborative agreement with PACT); and team member training in geriatrics. The survey questions with categorical answers were related to staffing, clinic space, other services provided in GeriPACT (e.g., use of patient educators, care managers, comprehensive geriatric assessments, and having a collaborative agreement with PACT), patient enrollment, patient conditions managed in the GeriPACT. The team member training questions’ answer category was on a 5-point Likert scale (scored 1 to 5: 1 = “no staff were trained” and 5 = “all staff were trained”). Both panel size and staff full-time employment equivalent (FTEE) were numeric variables. 

Model Consistency Summary Measure. In addition, we created a composite measure to examine how consistent VAMCs were to the GeriPACT model that is outlined in VA’s Handbook [7]. To assess this consistency, we assigned one point when each of the following model features was present. As such, each program could score between 0 and 5:

- Providers were assigned to a separate panel of patients and core GeriPACT staff members were assigned solely to the GeriPACT team. (1 point)
- GeriPACT teams had all staff assigned, including a provider, nurse, social worker, licensed practical nurse, clerical associate, social worker, and pharmacist/clinical pharmacist. (1 point)
- GeriPACT teams had dedicated clinic space either in PACT or Geriatrics. (1 point)
- Patient panel sizes were scaled to GeriPACT provider’s self-reported (MD and NP) FTEE (e.g., panel of 800 patients and 1.0 FTEE provider or panel of 400 patient and 0.5 FTEE provider). (1 point)
- GeriPACT served all Geriatric syndromes or conditions outlined in the Handbook. (1 point)

### 2.3. Data Collection

With support from the Office of Geriatrics and Extended Care, we identified 71 VAMCs nationwide with GeriPACT programs and received names of physician leaders at each site. Each physician leader received a personalized survey link sent via email. We fielded the web-based survey for 4 weeks in July 2016 and sent 3 participation reminders to non-responders. 

### 2.4. Data Analysis

We report frequencies for categorical measures and means with standard deviations for numerical measures (e.g., panel size and FTEE). To examine GeriPACT model consistency, we present frequencies for each individual model feature and then means and standard deviations for the composite measure which ranged from 0–5 points. All analyses were conducted using SAS version 9.2 (SAS Institute, Inc., Cary, NC, USA).

We analyzed the majority of structural characteristics data at the team level. Two structural characteristics were analyzed at the facility-level—data regarding “other” structures (e.g., use of patient educators, care managers, comprehensive geriatric assessments, and having a collaborative agreement with PACT) and team training in geriatrics. For these two structural characteristics, respondents were asked to think about all the GeriPACT teams at their facility rather than individual GeriPACT teams when answering these questions.

We analyzed the consistency with the GeriPACT model composite measure at the facility-level (i.e., across multiple GeriPACT teams). Thus, if any GeriPACT at the VAMC had a feature consistent with the GeriPACT model, they received one point regardless of the number of GeriPACTs present at the site. 

## 3. Results

The objective of our analysis was to evaluate the range and fidelity with which GeriPACT are implemented across VA by: (1) depicting data on individual GeriPACT structural characteristics; and (2) examining a composite measure to assess the extent to which medical centers were consistent with the GeriPACT model that is outlined in the VA’s Handbook. Physician leaders from 44 medical centers with at least one GeriPACT team participated in the survey (response rate of 62%). Within the 44 medical centers, there were 101 GeriPACT teams. In addition, 55% of the 44 medical centers had more than one GeriPACT team at their facility. Table 1 shows site and patient characteristics. The medical centers were geographically dispersed and 58% of medical centers were at VA’s highest level of complexity (1a). In Fiscal Year 2016, there were 32,408 unique GeriPACT patients who had 148,510 GeriPACT encounters. The average age of GeriPACT patients was 82.4 and average JEN facility index score was 4.30. 

Table 2 displays information on GeriPACT team composition and staff full-time employment equivalent. 

Disciplines on core teams: Our survey results indicated that GeriPACT core team members most often consisted of staff from the following disciplines: registered nurse, physician/geriatrician, social worker, clerical associate (i.e., medical support assistant), and the clinical associate (i.e., licensed practice nurse). Clinical pharmacists (24.8%) and nurse practitioners (18.8%) were least often included as GeriPACT core team members. 

Staff full-time employment equivalent: Physicians, nurse practitioners (when present on the team), and nurses (RN) had the highest mean FTEEs, while social workers and pharmacists had the lowest FTEEs. 

Table 3 and Table 4 display the distribution of GeriPACT structural and organizational characteristics for our sample. We found there was variation on all of the GeriPACT characteristics assessed. In addition, sites also varied on their resulting composite measure scores assessing consistency with the GeriPACT model.

Provider/Team assignment to GeriPACT: Three-quarters of the GeriPACT teams reported that providers were assigned a separate panel of patients and each panel shared core team members across various teams at the hospital. Fewer respondents reported other methods of assignment including providers being assigned their own panel of patients and each panel having other core team members assigned solely to the GeriPACT (12%), or providers sharing a panel of patients with other GeriPACT providers and having other core team members assigned solely to the GeriPACT (4%).

Space allocation: About two-thirds of GeriPACT teams had dedicated space either collocated in Geriatrics, Specialty Care, or Primary Care (with other PACT teams).

*GeriPACT patient assignment:* Respondents reported that PACT referrals (75%) and patient request (57%) were the methods most often used to assign patients onto GeriPACT panels. Patients assigned to GeriPACT teams included Veterans who were newly enrolled to VA and met the age criteria (49%), current PACT patients based on GeriPACT conditions (43%), and current PACT patients based on age (42%).

Other GeriPACT features: Physician leaders reported that 39% of GeriPACTs had written a collaborative service agreement with PACT. In addition, 72% of GeriPACTs had designated educators to provide patient education, and almost 69% of GeriPACTs had a designated case manager to coordinate across specialties or with providers outside of GeriPACT. Furthermore, 64% of physician leaders reported that they perform a Comprehensive Geriatric Evaluation as part of their GeriPACT work.

*Patients’ conditions served:* The majority of facilities with a GeriPACT provided care to patients with geriatric conditions listed in the Handbook (see Table 4). The conditions most often served were: psychosocial concerns, advanced age, disorders of gait and balance, experiencing multiple medical and functional concerns, and frailty.

Team members’ training: We found 78% of facilities with a GeriPACT reported that “most” or “some” team members were boarded or certified in geriatrics. For other team members where certification and boarding were not possible, roughly two-thirds of facilities reported that only “*a few*” or “*none*” of their staff had advanced formal training in Geriatrics.

Consistency with the GeriPACT Model expectations: Table 5 displays the features of the GeriPACT model and descriptive statistics regarding the summary score. We found that 45.5% of facilities had adequate panel size given provider FTEE, 43.2% had dedicated space, and 36.4% served 15 Geriatric conditions served in GeriPACT. The mean value of the composite measure was 2.03 with scores ranging from 1 to 4. About 6% of facilities were highly consistent (e.g., scoring 4 or greater on the summary measure) with the GeriPACT model as outlined in the VA’s Handbook.

## 4. Discussion

This study presented an overview of how VA Medical Centers have structured the GeriPACT model and explored the extent to which facilities were consistent with expected GeriPACT model features. Our results indicate that there was variation in these structural characteristics across GeriPACTs. For example, GeriPACTs recruited patients in different ways and not all programs had patient educators and care managers to assist with coordination with other service or had collaborative agreements in place with PACT.

There are several reasons that may explain why we found considerable variation in structural characteristics across GeriPACTs. The GeriPACT model was implemented at the facility-level in collaboration with local GeriPACT team leaders [8]. GeriPACTs were often developed from previous Geriatric Primary Care clinics; thus, some of their structural elements may have been carried over from when the clinic used to function differently, for example, as part of Specialty care. The process of setting up the GeriPACT could be collaborative and adaptive allowing some customization in program structure based on fit with the hospital environment and provider preferences [11]. In addition, implementation of the GeriPACT model may have received varying levels of support from service line and executive leaders at the VAMCs resulting in varying levels of resources provided for program implementation.

In addition, we observed that very few GeriPACTs (6%) were consistent with the GeriPACT model features that are outlined in the VA’s Handbook. In particular, there were two notable areas where additional resources may be needed to meet these model elements—GeriPACT staff and dedicated space to run the clinic. Often times, staffing a GeriPACT team at the appropriate levels can be difficult because of turnover. In addition, there is a national shortage of providers trained in geriatrics [12]. Furthermore, medical centers may lack resources (i.e., budget) to hire enough staff to cover more than one GeriPACT team at a facility. To further implement the model, more support will be required to bring in appropriate staff and space to serve older Veterans.

The GeriPACT model is evidence-based as it has been adapted from the VA’s Geriatric Primary Care model which emphasizes continuity and coordination of care that is focused on care planning and patient follow-up, inclusion of caregivers, and accessibility of the team [11]. These facets of care have been shown to have a positive effect on outcomes [13,14,15]. However, our results raise important questions including whether consistency to the model, or the presence or absence of GeriPACT features, has an impact on outcomes; and which of the features highlighted in this article are the most influential in improving process and patient outcomes.

Our study has several strengths worth highlighting. This is the first national evaluation of GeriPACT structural characteristics within the VA—a large integrated healthcare system. In addition, we had a relatively high survey response rate (62%) from GeriPACT physician leaders, who have the most comprehensive understanding of structural characteristics of their facility’s GeriPACT team(s). We also collaborated closely with our national operational partner, the VA Office of Geriatrics and Extended Care, to develop a focused survey that could be used to inform policy. Additionally, our findings may be applicable to other patient care models used within the VA, such as PACT, and outside the VA, such as PCMH.

However, there are some limitations. Although physician leaders are the most knowledgeable key informants, the data are self-reported which could result in some over or underestimation. In addition, our list of facilities with a GeriPACT was obtained from the National Program Director in April 2016; thus, we may have inadvertently omitted any GeriPACTs that formed closer to the time of survey administration in July 2016. Additionally, although our response rate was 62%, we are unable to report structural characteristics from GeriPACTs that chose not to participate. Thus, we may not be capturing the full range of structural characteristics for all GeriPACTs in VA.

## 5. Conclusions

In conclusion, our study findings provided important information about GeriPACT structural characteristics and model consistency that can be used to shape VA policy. In particular, results can be used to inform the GeriPACT handbook and operations manual. The VA may consider tailoring some of the GeriPACT structural elements or working with VAMCs to implement GeriPACTs as closely aligned to the features in the Handbook as possible. This is particularly important as we found many sites had not implemented all expected GeriPACT model features. Future research should focus on relating structural characteristics to care processes and patient outcomes. Given that GeriPACT is a newer care model in the VA that was tailored for older adults, assisting VAMCs in their journey to implement GeriPACT will help facilities to provide intensive and coordinated patient-centered care for older patients through a specialized multi-disciplinary team. These results are also applicable to the private sector, as many healthcare systems are consolidating into larger integrated networks becoming more similar to the VA.

## Figures and Tables

**Table 1:** Site and Patient Characteristics in FY2016 (N = 44).

Site Characteristic	Percent			
VA Complexity ^1^				
1a-Highest Complexity	58.1			
1b-High Complexity	23.2			
1c Mid-High Complexity	9.3			
2-Medium Complexity	4.7			
3-Low Complexity	4.7			
Region				
Northeast	20.5			
South	25			
Midwest	29.5			
West	20.5			
Outside Continental US	4.5			
	**Mean**	**(SD)**	**Min**	**Max**
Number GeriPACT encounters	3453.7	(3510.1)	35	14,782
Unique Number GeriPACT Patients	753.7	(679.6)	33	3206
GeriPACT Patient Age	82.4	(8.4)	59	106
GeriPACT Patient JEN Frailty Index	4.3	(2.3)	0	12

^1^ Facilities are categorized into one of five groups: 1a (most complex), 1b, 1c, 2, and 3 (least complex). Because the facility complexity model uses indexes of multiple variables, there is no formula that defines what qualifies as a 1a facility, 1b facility and so on. Instead, VA generally categorizes the types of facilities that are in each facility complexity group based on their facility and program characteristics such as volume, high risk patients, mix of clinical programs, and types of research and teaching programs.

**Table 2:** GeriPACT Staff FTEE and Panel Size (N = 101).

FTEE	N	Mean	SD	Min	Max
Physician	57	0.626	0.432	0.080	2.00
Nurse Practitioner	24	0.683	0.455	0.100	2.00
Nurse Care Manager	65	0.524	0.397	0.025	1.30
Social Worker	67	0.297	0.289	0.025	1.00
Clinical Associate	48	0.483	0.441	0.025	2.00
Administrative Associate	49	0.447	0.338	0.050	1.00
Pharmacist	46	0.256	0.287	0.010	1.00
**Panel Size**					
Maximum ^1^	81	394.667	362.608	0	2053.00
Average ^2^	81	300.519	282.768	0	1369.00

^1^ Maximum panel size is the maximum number of patients allocated to the team’s panel. ^2^ Average panel size is the typical number of patients on a team’s panel.

**Table 3:** GeriPACT Structural and Organizational Characteristics (N = 101).

Structural Characteristic	Percent
**Provider/Team Assignment**	
Providers assigned separate panel of patients and each panel shares core team members	76
Providers assigned separate panel of patients and each panel has its distinct core team members	12
Providers share panel of patients and panel has its own core members	4
Other	8
**Space**	**Percent**
Dedicated space collocated in Geriatrics Specialty Care	33
Collocated space in PACT	25
Does not have dedicated space and shares space with other clinics	25
Other	6
**Patient Assignment**	**Percent**
By PACT Referral Only	75
By Patient Request	57
Only Newly Enrolled to VA: Aged-Based	49
Current PACT patients: Condition-focused	43
Current PACT patients: Aged Based	42
Only Newly Enrolled to VA: Condition-based	21
**Other Structures**	**Percent**
Report Comprehensive Geriatric Evaluation	64
Written Collaborative Service Agreement with PACT	39
Designated case manager to coordinate across specialties/providers outside GeriPACT	69
Designated educators	72

**Table 4:** Patient Conditions served in GeriPACT (N = 101).

Conditions Served	Percent
Psychosocial concerns	81.4
Advanced age	80.4
Disorders of gait and balance	80.4
Multiple medical and functional concerns	79.4
Frailty	79.4
Falls	78.4
Incontinence of bowel and bladder	78.4
Dementia and other causes of impaired cognition	78.4
Depression	78.4
Risk for institutionalization placement or concern about independence in living arrangements	78.4
Failure to thrive	77.5
Elder abuse/neglect	77.5
Delirium	74.5
Impending disability	73.5
Documentation of suboptimal outcomes in PACT	55.9

**Table 5:** Consistency to the GeriPACT Model (N = 44).

Features Consistent with GeriPACT Model	Percent			
appropriate panel size and allocation of GeriPACT provider	45.5			
dedicated space and location in PACT or Geriatrics	43.2			
serving all appropriate medical conditions	36.4			
providers were assigned to separate panels with distinct core members	11.4			
composition of the core and extended team included appropriate staff	11.4			
**Summary Measure**	**Mean**	**(SD)**	**Min**	**Max**
Consistency to GeriPACT Model	2.03	(0.89)	1	4

## Supplementary Materials

The following are available online at http://www.mdpi.com/2308-3417/3/3/46/s1.

## Previous Dissemination

Data on staffing and FTEE were presented in a different format (i.e., at the facility-level) in a Journal of American Board of Family Medicine article. Data on GeriPACT model were presented at a 2017 Annual AcademyHealth meeting poster session (Adjognon).
