# Supplementary material for: Geriatric Patient-Aligned Care Teams in Department of Veterans Affairs: How Are They Structured?
Source: Geriatrics (Basel). 2018 Aug 1;3(3):46. doi: 10.3390/geriatrics3030046 (PMC6196730; doi:10.3390/geriatrics3030046)
Supplement: Supplementary file 1 [file geriatrics-03-00046-s001.pdf]

How VA Provides Care to Veterans in GeriPACT: A Survey of GeriPACT Physician Leaders

How would you describe the structure of your GeriPACT team?

- ☐ Providers are assigned a separate panel of GeriPACT patients and each panel has its own distinct GeriPACT core team members (e.g., social worker, nurse care manager, pharmacist).
- ☐ Providers are assigned a separate panel of GeriPACT patients and each panel shares core GeriPACT team members (e.g., social worker, nurse care manager, pharmacist).
- ☐ Providers share a panel of GeriPACT patients and the panel has its own distinct GeriPACT core team members (e.g., social worker, nurse care manager, pharmacist).
- ☐ Other Specify: ________________________________

What type of dedicated space does your GeriPACT have to see patients?

☐ We do not have dedicated space and share space with other clinics

☐ We have dedicated space co-located within PACT

If yes: How many dedicated rooms do you have to see patients? ____

☐ We have dedicated space co-located in Geriatrics specialty care

If yes: How many dedicated rooms do you have to see patients? ____

> A GeriPACT teamlet is a primary care team that generally consists of a **core team**, which may include a provider (Physician, Advance Practice Nurse (APN), Physician Assistant (PA)), Residents in training, Registered Nurse (RN) Care Manager, Clinical Associate (Licensed Practical Nurse (LPN) or Medical Assistant/Health Technician), Social Workwe, Clinical pharmacist, and an Administrative Associate (Clerk/Health Technician).
>
> An **extended team member** of GeriPACT is a health care professional designated to a GeriPACT/PACT position in Primary Care Management Module (PCMM) who provides direct discipline-specific patient care to one or more panels of patients, but not to all primary care patients at the facility. *Examples of extended team members are*: Registered Dietitians and Primary Care-Mental Health Integration staff.

Which disciplines participate in the GeriPACT core and extended team?

☐ Audiologist
☐ Chaplain
☐ Clerical Associate/Receptionist (e.g., MSA)
☐ Clinical Pharmacist/ Clinical Pharmacist Specialist
☐ Clinical RN Specialist
☐ Dentist/Dentist Auxiliary
☐ Dietician
☐ Health Administrator
☐ Kinesiotherapist/KT Aide
☐ Licensed Practical Nurse
☐ Nurse Aide
☐ Nurse Practitioner
☐ Occupational Therapist/OT Aide
☐ Physical Therapist/ PT Aide
☐ Physician Assistant
☐ Physician/Geriatrician (e.g., MD, DO)
☐ Psychiatrist/Geropsychiatrist
☐ Psychologist
☐ RN (e.g., Nurse Case Manager)
☐ Social Worker
☐ Speech or Language Pathologist
☐ Other Specify: ______________________

Enter the assigned GeriPACT FTEE each of GeriPACT core and extended team members.

	FTEE (0-1)
[insert disciplines]	

What is the maximum panel size of your GeriPACT in Fiscal Year (FY) 2016? (i.e., the largest number of patients in panel you can accommodate)

____

Through which of these processes are patients assigned to the GeriPACT panel?
Choose all that apply.

☐ Only Newly Enrolled patients to VA: Age based enrollment
☐ Only Newly Enrolled patients to VA: Condition focused enrollment
☐ Current PACT patients: Age based enrollment
☐ Current PACT patients: Condition focused enrollment
☐ By PACT referral
☐ By patient request

Which conditions or concerns are managed by the GeriPACT team? Choose all that apply.

	Yes	No
Advanced age	☐	☐
Multiple medical and functional concerns	☐	☐
Psychosocial concerns	☐	☐
Failure to thrive	☐	☐
Frailty	☐	☐
Delirium	☐	☐
Falls	☐	☐
Disorders of gait and balance	☐	☐
Incontinence of bowel and bladder	☐	☐
Dementia and other causes of impaired cognition	☐	☐
Depression	☐	☐
Documentation of suboptimal outcomes in PACT (high utilization)	☐	☐
Elder abuse / neglect	☐	☐
Risk for institutional placement or concern about independence in living arrangements	☐	☐
Impending disability	☐	☐
Other: Specify: ________________	☐	☐

	Yes	No
Do you report the procedure code for Comprehensive Geriatric Evaluation (i.e., S0250) as part of the activity of this GeriPACT?	❑	❑
Does your VAMC have a written collaborative service agreement between PACT and GeriPACT concerning proposed transfer of patients?	❑	❑
Do you have a specified person(s) (e.g. case manager) in your clinic, other than a physician, NP, or PA, who works to arrange and coordinate care across specialties and with providers other than those in the GeriPACT clinic?	❑	❑
Do you have designated educators, other than a physician, NP, or PA, available in your clinic to teach patients how to self-manage their illnesses and medication regimens?	❑	❑

	Most	Some	A Few	None
How many GeriPACT team members are certified /boarded within their disciplines?	❑	❑	❑	❑
Other than those above, do you have team members with advanced formal training in their discipline (e.g., curriculum in geriatric education center, a one month or longer clinical mentored preceptorship)?	❑	❑	❑	❑
